# Preimplantation Genetic Testing for Genetic Diseases: Limits and Review of Current Literature

**DOI:** 10.3390/genes14112095

**Published:** 2023-11-17

**Authors:** Roberta Giuliano, Anna Maione, Angela Vallefuoco, Ugo Sorrentino, Daniela Zuccarello

**Affiliations:** 1 Preimplantation Genetic Diagnosis, Department of Women’s and Children’s Health, University of Padova, 35128 Padova, Italy; 2 Fertility Unit, Maternal-Child Department, AOU Federico II, 80131 Naples, Italy; rosymaio@hotmail.it; 3 Department of Clinical Medicine and Surgery, University of Naples Federico II, 80138 Naples, Italy; angela.vallefuoc9@gmail.com; 4 Clinical Genetics and Epidemiology Unit, University Hospital of Padova, Via Giustiniani 3, 35128 Padova, Italy; ugo.sorrentino@unipd.it (U.S.); daniela.zuccarello@unipd.it (D.Z.)

**Keywords:** preimplantation genetic testing, biopsy, pgt-m, pgt-a, pgt-sr, limits

## Abstract

Preimplantation genetic testing (PGT) has emerged as a revolutionary technique in the field of reproductive medicine, allowing for the selection and transfer of healthy embryos, thus reducing the risk of transmitting genetic diseases. However, despite remarkable advancements, the implementation of PGT faces a series of limitations and challenges that require careful consideration. This review aims to foster a comprehensive reflection on the constraints of preimplantation genetic diagnosis, encouraging a broader discussion about its utility and implications. The objective is to inform and guide medical professionals, patients, and society overall in the conscious and responsible adoption of this innovative technology, taking into account its potential benefits and the ethical and practical challenges that it presents.

## 1. Introduction

Preimplantation genetic testing (PGT) is a diagnostic procedure that allows the analysis of embryos during the initial stages of development, enabling the selection and transfer of healthy ones into the maternal uterus, thereby reducing the risk of transmitting severe genetic disorders and enhancing the likelihood of a successful pregnancy. Moreover, it empowers couples to avoid the challenging decision of pregnancy termination in cases of embryos affected by serious genetic conditions. PGT techniques are widely used in clinical practice today and, at the same time, represent an area of significant interest, including commercial, in terms of scientific and technological research. PGT involves a multidisciplinary approach, as it is inseparable from medically assisted reproduction (MAR) techniques and facilitates the investigation of monogenic hereditary disorders (PGT-M) and numerical (PGT-A) and/or structural (PGT-SR) chromosomal abnormalities. This procedure originated in the 1990s with methods based on polymerase chain reaction (PCR), and, since then, it has developed over time with the implementation of various strategies. Such evolution has accelerated in recent years, in which molecular biology techniques applied to laboratory diagnostics have been characterized by robust technological advancements, allowing for the analysis of an increasing number of molecular targets while significantly reducing analysis times and costs.

**Figure 1:**
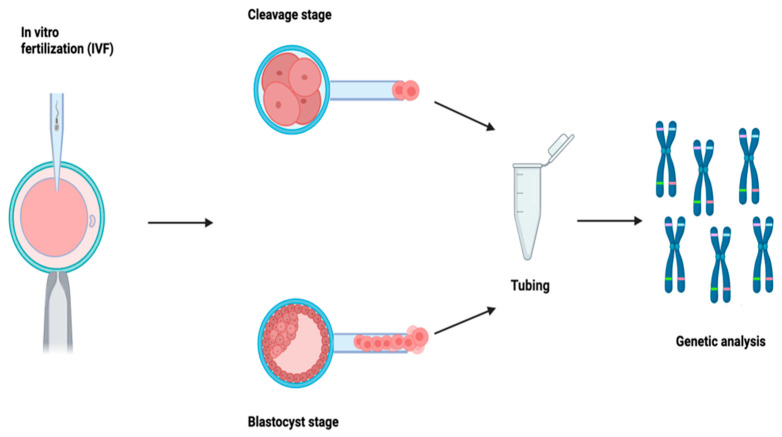
After an assisted fertilization cycle, a biopsy is performed, and the sample of extracted cells is then subjected to genetic analysis to determine whether the embryo is suitable for transfer or not.

In particular, recent advancements in human genome sequencing have led to the development of next-generation sequencing (NGS) technologies, which currently represent the gold standard for the screening of selected gene panels or extensive portions of the genome. However, the vast amount of data produced by these technologies can provide genetic information that is often challenging to interpret within a specific clinical context. Hence, despite NGS techniques surpassing traditional molecular analysis methods in terms of technological prowess, the use of the latter remains relevant in the investigation of known molecular targets, such as searching for a familial mutation or viral genome. Therefore, while it is true that technological advancements have rendered diagnostic strategies increasingly efficient, maintaining diverse reference strategies remains a superior approach. This is because some strategies might possess efficiency limitations that necessitate the implementation of custom analysis protocols tailored to the clinical case. In order to offer a comprehensive perspective to professionals and patients, as members of the Italian Network of Preimplantation Genetic Testing (N.I.D.O.), in this narrative review, we aim to address the limitations, potential, and applications of past and current PGT techniques (Figure 1).

## 2. Biopsy Techniques

Embryonic biopsy plays a pivotal role in the ART process and in the prevention of hereditary genetic diseases. Embryos generated using assisted reproductive technologies (ART) from couples undergoing/accessing preimplantation genetic diagnosis are initially subjected to biopsy procedures to obtain an adequate number of cells for genetic screening. Genetic analysis can be performed on one of the following sample types: polar bodies (PB), blastomeres (collected during the cleavage stage), or trophectoderm cells (TE). Each of these procedures has inherent limitations and requires the opening of the zona pellucida (ZP). Over the years, various approaches have been employed, ranging from mechanical to chemical methods, culminating in the currently predominantly used laser-assisted approach. This involves the use of a laser to open the ZP, followed by the aspiration of cells using a micropipette for biopsy purposes.

Polar bodies (PBs) are products of female gametogenesis, i.e., oogenesis, not significant from a reproductive perspective, as they cannot be fertilized. PB biopsy can be performed either simultaneously, where both PBs are collected together 16 h after insemination, or sequentially, where the first PB is retrieved before insemination (4 h after oocyte retrieval) and the second after fertilization (16–18 h after insemination). The single-step approach appears more advantageous as it allows only fertilized oocytes to be analyzed, reducing analysis time and costs. While PB biopsy is the less invasive approach, on the other hand, it only provides information mainly related to maternal genetic contribution, maternal-origin meiotic aneuploidies, or maternal pathogenic variants [1]. This strategy, although outdated according to current best practices, remains available in countries with legal restrictions on genetic embryo evaluation and cryopreservation.

Regarding the collection of the sample from the blastomere, involving a biopsy at the cleavage stage, its main benefit would be that a biopsy performed at this stage would enable fresh transfers, as many modern genetic techniques can provide results within 24 h of sample receipt. However, despite overcoming the limitations observed with PB biopsies, it is worth considering that, in this case, only one to two blastomeres can be retrieved. This is because the embryo at this stage consists of 6–8 cells; therefore, taking more cells would result in the depletion of about 30% of the embryonic mass, consequently affecting its viability [2]. Furthermore, analyzing 1–2 cells does not allow the detection of potential mosaicism, representing another significant limitation of embryo biopsy at the cleavage stage.

In the case of a blastocyst on the 5th/6th day of development, the main advantage lies in the larger amount of DNA available for analysis. Since a blastocyst consists of at least a hundred cells, more cells can be retrieved compared to an embryo on the 3rd day (5–10 cells). This also increases the chances of detecting the presence of common mosaicisms in blastocysts [3,4]. Another advantage is that cells are taken from the trophectoderm, which is not directly involved in fetal formation, as opposed to inner cell mass (ICM) cells. This makes the procedure less invasive for embryonic development compared to a biopsy on the 3rd day. Furthermore, due to the larger amount of analyzed DNA, the likelihood of inconclusive diagnoses is estimated to be less than 5%, and multiple analyses for different indications can be performed from the same sample after whole-genome amplification (WGA) [5]. Finally, some studies have demonstrated that embryos vitrified at this stage exhibit a higher survival rate compared to embryos at the cleavage stage. This allows for postponed transfer and the execution of single embryo transfer (SET) to prevent multiple pregnancies [6].

### Timing of Biopsy

The timing of biopsy is a highly debated topic and is crucial to ensure accurate and reliable results in genetic analysis. The first approach involves creating a laser-incised hole in the ZP on day 3 and then waiting for the herniation of the blastocyst on day 5/7 [7]; however, for embryos that are slightly slower in development, it is preferred to wait one more day and then perform the biopsy on the 4th day [8]. Although widely adopted, this procedure carries the risk of herniating the ICM, and the embryo undergoes two manipulations outside the incubator. The second approach extends blastocyst culture to the stage of complete expansion. This allows simultaneous ZP opening and TE cell retrieval, while intervening only once on the embryo and opening the ZP far from the ICM [9]. The third approach combines the aforementioned methods, opening the ZP when the blastocyst is fully expanded and waiting for natural herniation. Patrizia Rubino et al., 2020, [10] demonstrated that the choice of biopsy protocol could impact clinical outcomes. Specifically, ZP opening during the blastocyst stage with simultaneous biopsy results in a higher survival rate after thawing compared to the approach that involves ZP opening during the cleavage stage [10]. Despite the limitations, embryonic biopsy remains the only tool for couples seeking to have healthy children and to prevent hereditary genetic diseases. Ongoing research and technological progress in this field could help to overcome some of the current limitations and further enhance the safety and efficacy of embryonic biopsy in ART. Following the biopsy, cells are washed and placed in a tube before being sent to the genetic laboratory for analysis.

## 3. Preimplantation Genetic Testing for Monogenic Disorders (PGT-M)

Preimplantation genetic tests have a broad range of applications, conceptually divisible into two main areas: inherited disorders, where alterations can be found in the parents (PGT-M and PGT-SR), and de novo conditions, i.e., not inherited, as in the case of PGT-A.

The objective of PGT-M testing is to avoid transferring embryos affected by a specific monogenic disease. This can only be achieved by selecting embryos that either do not carry the mutation or are healthy carriers (in the case of recessive diseases), as may occur in patients with a positive family or personal history for a monogenic condition. This necessitates a preliminary study tailored to each couple, involving family members.

In general, PGT-M can be applied to the diagnosis of all hereditary monogenic diseases for which the responsible gene has been identified, one or two index cases are available, and a diagnostic linkage analysis protocol can be developed. Conversely, it is not indicated in cases of large gene deletions/duplications or de novo triplet expansions since the phasing of the at-risk haplotype is not feasible.

An exceptional application of PGT-M is HLA typing, which aims to select a HLA-compatible embryo for an affected sibling in need of a hematopoietic stem cell transplant [5,6,7,8,9,10,11]. However, embryo selection based on HLA typing has sparked numerous ethical debates over the years, as well as the application of PGT in diseases that affect people when they are adults (for instance, Huntington’s disease). In fact, for late-onset diseases, it is usually expected that the at-risk partner of the couple undergoing PGT has already undergone presymptomatic testing. However, in most cases, the at-risk partner may not wish to know their own genetic status but still be concerned about transmitting the mutation to their child. In these cases, one can either proceed with exclusion testing, thus avoiding presymptomatic testing for the at-risk partner, or perform PGT simultaneously with the analysis of embryonic aneuploidies, testing the at-risk partner without specific result disclosure (non-disclosure test).

Nevertheless, despite significant advancements and promising prospects, PGT-M presents challenges and limitations that necessitate a thorough analysis to optimize its clinical efficacy and address ethical and practical concerns.

### 3.1. Diagnostic Strategies

PGT-M always starts with the biopsy of the TE, from which 5–10 cells are collected. From there, the process can split into either target amplification or whole-genome amplification [12]. In the case of target amplification, a multiplex PCR can be performed, identifying both the mutation and genetic markers. With whole-genome amplification, options include multiplex PCR, single nucleotide polymorphism (SNP) array, or NGS. All these techniques are based on the principle of haplotyping, which involves determining the risk-associated haplotype linked to the mutation by observing how chosen informative markers (SNPs or STRs) segregate with the mutation. This approach helps to determine whether the embryo has inherited the risk allele or the wild-type allele. This is a genuine linkage analysis, which overcomes the limitations of working with low DNA quantities. Therefore, during preclinical work-up, the genotyping of SNP markers near the gene of interest in DNA samples from the couple and family members with a known genetic status is required [13,14].

PGT-M originated from fragment length analysis using STRs, combined with minisequencing for mutation analysis. In parallel, another target technology using SNPs with real-time PCR was developed, enabling the identification of mutations and flanking SNP markers. The workflow involves real-time PCR SNP genotyping, TE biopsy and the lysis of collected cells, multiplex amplification using TaqMan assays for mutations and informative SNPs, real-time PCR amplification, TaqMan genotyping, linkage analysis, and haplotype determination. If CNV assays are added to TaqMan assays in the second step, the simultaneous detection of aneuploidies (PGT-M and PGT-A) is also possible.

Among the advantages of the target protocol is a very low allele drop out (ADO) rate, which increases with WGA instead. Additionally, diagnoses can be obtained quickly (within 4 h of biopsy) due to the short analysis time of real-time PCR compared to NGS. Concurrently, WGA started to develop, allowing the amplification of the entire genome with multiple displacement amplification (MDA), followed by the target amplification of STR markers and fragment length analysis. Following the introduction of WGA, PGT-M approaches diversified greatly. With target amplification, specific loci or gene regions are amplified, whereas, with WGA, the entire genome is amplified. This opens up the possibility to use other techniques, such as SNP array, especially karyomapping, suitable for PGT-M; targeted NGS, enriching specific gene regions linked to particular pathologies; and genome-wide NGS, with an enriched panel (5000 genes) to detect various pathologies [15]. The advantages of SNP array are the standardized procedures and reduced time to diagnosis, allowing concurrent PGT-M, PGT-A, and PGT-SR. However, there is a drawback in terms of higher costs and the analysis being limited to inherited pathologies, excluding de novo mutations due to the need for a reference. In particular, SNP genotyping methods are preferable to STRs for indirect analysis, as SNPs are more frequent in the genome compared to STRs and thus more informative in this context. Therefore, whenever possible, SNP analysis should be preferred over STR analysis [16]. The diagnostic protocol of the targeted strategy involves the direct identification of the disease-causing mutation post-WGA, along with analyzing at least two informative polymorphic markers, or three markers in cases where the causative mutation is unknown [17]. The relevant polymorphic markers are STRs, chosen for their informativeness. An informative single STR is equivalent to three informative SNPs, even though SNPs (biallelic) are more abundant (one SNP every 300–1000 bp), easier to interpret, and suitable for high-throughput analysis [14]. Subsequently, a multiplex PCR is set up using pre-made kits, and the heterozygosity of STRs is assessed via capillary electrophoresis. Since markers are chosen based on the chromosome affected by the mutation, those close to the gene of interest allow the discrimination of parental haplotypes. Informative markers (heterozygous) are selected for clinical testing on embryos. High-risk (mutant) and low-risk haplotypes are established during preclinical setup. The diagnostic protocol is optimized for the couple and family members to confirm the presence of the causative variant and define informative linked markers for PGT [18]. As a result, PGT-M is a laborious and costly procedure that requires extensive preliminary family study, leading to long waiting lists for couples embarking on this journey. This approach also enables molecular diagnosis while simultaneously testing for DNA contamination, relatedness, and technical artifacts [19,20].

### 3.2. PGT-M Limitations

One of the main limitations of PGT-M is the genetic variability and complexity inherent to monogenic diseases themselves. Hereditary genetic diseases can be caused by different mutations on the same gene or mutations on different genes that lead to similar phenotypes. This genetic variability can make the accurate identification of specific disease-causing mutations challenging. Moreover, certain genetic diseases can be influenced by environmental or epigenetic factors, further complicating the association between genotype and phenotype. The challenge in addressing genetic variability in monogenic disorders is that a preimplantation diagnostic test should accurately identify the presence or absence of specific mutations in embryonic cells. The current clinical practice in PGT centers does not involve the preimplantation diagnosis of VUS. However, the array of mutations and the heterogeneity of phenotypes can impact the efficacy of such tests. Furthermore, the detection of Variants of Uncertain Significance (VUS) can further complicate result interpretation, generating uncertainty about which mutations actually represent a concrete risk in terms of developing the disease. Dealing with genetic variability and the complexity of monogenic disorders requires a combination of advanced molecular, diagnostic, and bioinformatic approaches. Identifying specific genetic variants responsible for a disease requires a deep understanding of its genetic basis and the metabolic pathways involved. Additionally, collaboration between clinical geneticists, molecular biologists, and bioinformaticians is crucial in interpreting genetic information correctly and identifying relevant mutations for PGT-M.

Another major technical limitation of PGT-M protocols is the small quantity of input samples, which is why WGA is performed following tubing and cell lysis in order to obtain a sufficient quantity of genomic DNA for one or more molecular analyses [20,21]. As mentioned earlier, the gold standard for PGT-M involves an indirect analysis because the risk of allele drop out (ADO), amplification failure (AF), and contamination is very high when working with embryos, as only a few cells are available for the examination. Moreover, determining the haplotype through SNP array also requires a sample from at least a first-degree relative of the carrier partner. If unavailable, this leads to reduced SNP informativeness. Conversely, this limitation can be mitigated with a targeted protocol, although it is more time-intensive compared to NGS, which provides a greater quantity of information in less time.

Certain mutations (e.g., small deletions, duplications, or complex chromosomal rearrangements) cannot be detected and diagnosed using current analytical strategies. This has led to the introduction of new screening approaches capable of analyzing genetic markers present throughout the genome (genome-wide), thereby overcoming these limitations. The use of WGA has made it possible to apply genome-wide analysis methods to samples consisting of single or few cells [22,23]. An example is karyomapping, which is based on SNP array technology and determines an individual’s genotype by analyzing thousands of SNPs distributed across the genome. Each SNP is biallelic, presenting one of two possible nucleotides on each chromosome. Individually, they are less informative than STRs, which possess high allelic heterogeneity. However, a set of four SNP markers is sufficient to determine the genotypes of the parents and the index case [24]. Karyomapping is based on linkage analysis: by comparing the SNPs associated with the disease-causing mutation in the index case and parental chromosomes with those present in embryo cells, it is possible to identify the presence or absence of the mutation-carrying allele. Therefore, karyomapping marks a transition from a family- or disease-specific diagnostic approach to a “genome-wide” approach, applicable, in principle, to any monogenic alteration with informative SNPs. Moreover, this technique overcomes the issue of failed allele amplification in single-cell cases, distinguishing key SNPs in the embryo sample from non-key SNPs. This does not completely eliminate genotyping errors but significantly reduces their occurrence and, in most cases, leads to the identification of a set of key SNP markers with consistent results.

### 3.3. How Does Karyomapping Work?

SNP array is a highly dense array spotted with 300,000 SNPs. Firstly, the parents and a reference individual need to be genotyped—collectively called a trio—across hundreds of thousands of sites distributed throughout the genome. This is because karyomapping is based on genome-wide linkage analysis, targeting all 300,000 available platform SNPs. The first step identifies a set of informative SNP markers (heterozygous for one parent and homozygous for the other) for each of the four parental chromosomes. Subsequently, the allele phase for each informative SNP locus is determined, and the linkage of the parental risk alleles with the corresponding chromosomes is established. A linkage is defined with respect to the reference individual’s genotype, usually an individual with a known disease state, such as an affected child or fetus from a previous pregnancy. The goal is to determine the parental origin of each chromosome in the embryo with regard to the reference genotype.

The first publication concerning karyomapping technology dates back to 2010 and was authored by Professor Handyside, the father of PGD, and colleagues [24]. This publication summarizes the key characteristics and execution of PGT-M karyomapping technology. Firstly, it is a genome-wide linkage analysis, a distinct advantage from certain viewpoints as it is performed on an extensive, non-target set of markers—specifically, all 300,000 available on the platform. Another distinguishing feature is its applicability at the single-cell level, necessitating a whole-genome amplification step. 

Furthermore, unlike other methods, no patient-, family-, or disease-specific setup is required. In 2014, Natesan et al., 2014, [25] conducted a concordance analysis, assessing karyomapping’s accuracy against the gold standard at that time—direct mutation analysis plus linkage analysis with flanking STR markers. This study involved 218 embryo samples from 44 PGD cycles, achieving an extremely high concordance rate of 97.7%. Discrepancies mainly arose in consanguineous families where, without direct mutation analysis, distinguishing the inheritance of the four alleles solely from haplotypes became more challenging. Another advantage is that, as no preclinical work-up is necessary, waiting times for setup, the potentially necessary acquisition of locus-specific probes, and validation on parents and family members are significantly reduced [25]. The WGA technology used in SNP array is multiple displacement amplification (MDA). This involves isothermal whole-genome amplification utilizing random hexamer molecules that bind to denatured DNA strands, continuously extending on the nascent strand, assisted by a phi polymerase. This generates large fragments and maintains high sequencing quality due to minimal biases. This feature is unique to karyomapping, as it is not shared by other techniques like NGS [26].

In conclusion, one of karyomapping’s advantages is the substantial reduction in laboratory workload and waiting times for couples due to the absence of the need for preclinical work-up. Additionally, it enables the simultaneous analysis of PGT-M and PGT-A since both SNP genotyping and chromosome copy number information are obtained from raw data. Disadvantages include the high costs of equipment and consumables, the underlying algorithm not providing an all-in-one solution for molecular and chromosomal diagnosis, being limited to inherited chromosomal or monogenic abnormalities only, and the necessity of pertinent familial samples for haplotyping. Lastly, direct mutation analysis is not included in this approach.

### 3.4. Future Perspectives for PGT-M

Improving diagnostic capabilities and accessibility for PGT-M is a key direction for the future. This includes addressing challenges associated with de novo mutations or mutations that are still difficult to identify, such as repeat expansions. New technologies like haplotyping-by-sequencing offer the potential to obtain both genetic and chromosomal information in a single workflow. However, it is important to note that this approach still requires family members for phasing, and, currently, it may not be suitable for the detection of de novo mutations.

## 4. Preimplantation Genetic Testing for Aneuploidy (PGT-A)

PGT-A is indicated for couples with advanced maternal age, recurrent implantation failure, and recurrent spontaneous abortions. It aims to identify any numerical chromosomal abnormalities (aneuploidies) in embryos. The selection of the best embryo is often based on morphological evaluation, as it is an inexpensive, rapid, and non-invasive technique [27]. However, despite its widespread use, this technique is not proficient in detecting genetic abnormalities. To improve live birth rates with the transfer of a single embryo, the use of preimplantation genetic testing for aneuploidy (PGT-A) has significantly increased. Aneuploidy, often resulting from meiotic non-disjunction, with trisomy being the most common form, has led to PGT-A being considered a definitive tool for embryo selection based on euploidy [28]. The complex nature of PGT-A includes demanding biopsy procedures and subsequent genetic analysis. Performing biopsies during the cleavage stage may entail the risk of fewer embryos reaching the blastocyst stage [29].

The origins of PGT-A techniques (e.g., FISH) date back to the 1990s, when the first methods for the genetic analysis of embryonic cells were developed. However, during these early years, technology and genetic knowledge were still developing, limiting the accuracy in identifying chromosomal abnormalities. FISH, mainly used to screen common chromosomal aneuploidies involving chromosomes 13, 18, 21, X, and Y, utilizes chromosome-specific probes to determine the copy number of a given chromosome, or specific chromosomal regions, within the nucleus. The use of different fluorochromes allows specific probes to be marked, enabling the simultaneous visualization of different chromosomal targets within the same nucleus [30]. Centromeric-specific probes help to characterize chromosomes by defining their origin, while whole-chromosome-specific painting probes are used to characterize and define breakpoints of both balanced and unbalanced rearrangements [31]. However, due to the limitations of FISH analysis, embryonic biopsy techniques, and the high prevalence of mosaicism at this developmental stage, this technique did not consistently yield the desired results to improve pregnancy outcomes through assisted reproductive techniques.

Over the subsequent decades, advancements in genomics and molecular biology led to the rapid evolution of PGT-A methods, leading to the historical period of PGT-A 2.0 (2008–2012). During this period, the emergence of array CGH (aCGH) and SNP array marked a transitional phase where a different sample collection method was adopted, primarily focused on trophectoderm (TE) biopsy. This multicellular sample improved the diagnostic reliability. Technologically, this era represented a significant advancement by enabling the simultaneous analysis of all 24 chromosomes, a capability that the previous FISH methods lacked.

Array comparative genomic hybridization (aCGH) is a technique that, to date, has been somewhat surpassed by other methodologies. It is based on the fluorescent labeling of the DNA from the biopsied sample and reference DNA. These samples are co-hybridized on an array platform where probes with known sequences are spotted. These probes can be sourced from bacterial artificial chromosomes (BACs) or synthetic oligonucleotides specifically designed to cover certain gene regions. The workflow involves the detection of the fluorescent signal resulting from the co-hybridization of the two DNAs. The primary advantage, compared to previous techniques, is that the detection is automated through analysis software across the entire chromosome. Consequently, the key advantage of aCGH over FISH is the ability to cover the entire chromosome and all 24 chromosomes. The laboratory protocol involves whole-genome amplification as the initial step, as is the case with many other technologies, such as SNP array and NGS. The resolution limit, which is the smallest detectable fragment, is heavily dependent on the number of probes available across the chromosomes and the distance between each probe: for aCGH, the resolution limit in PGT is approximately 5–7 Mb. During the historical period in which aCGH was developed, it found extensive application and was utilized in numerous publications. In particular, a study by Capalbo et al., 2015, [32] compared aCGH and quantitative real-time PCR, both emerging during a period of significant technological advancement and both capable of screening all 24 chromosomes. From this study, involving the double biopsy and parallel analysis of 161 blastocysts using both technologies, 99.4% concordance was observed (remarkable, considering their differing protocols and underlying principles), with discordance of 0.6%. Consequently, they conducted a third technology validation study using SNP array for the discordant cases. Upon re-analyzing the biopsies of these embryos, they found that the reasons for the discordance were mainly associated with limitations pertaining to aCGH. These limitations include, first of all, the absence of chromosome-specific cut-offs because there is simply a balance of the two fluorescent signals, so aneuploidy cannot be modeled specifically for each chromosome; the WGA can also introduce amplification biases, which is why chromosome-specific cut-offs would be useful to establish the boundary between euploidy and aneuploidy for each chromosome.

In conclusion, aCGH enables the analysis of all 24 chromosomes, featuring automated, accurate, and shorter protocols compared to FISH. It has a good resolution limit (6–10 Mb), rendering it suitable for PGT-SR. However, its disadvantages include high costs, an inability to detect polyploidy, and a low resolution for mosaicism due to the absence of chromosome-specific cut-offs.

During the same historical period, SNP array technologies were also developed. An array is a high-density matrix containing up to several million probes, which allows the genotyping of hundreds of thousands of SNPs across all chromosomes. These SNPs are selected markers. Thus, a specific platform has a preset number of SNP markers, and, therefore, the position and number of these SNPs within the region of interest are fixed. Among the applications of SNP array, in addition to the previously mentioned ones in the PGT-M setting, there is chromosomal analysis. Extra or missing chromosomes can also be detected through genotyping data. Theoretically, monosomy and trisomy present distinct profiles in terms of allelic ratio values between the reference and alternative alleles. This is referred to as “B-allele frequency”. Hence, these methods have allowed a more comprehensive evaluation of embryonic chromosomal profiles, enabling the detection of aneuploidies more accurately and thoroughly. Their introduction has significantly improved the ability to identify subtle quantitative chromosomal alterations that were not easily detectable using previous methods. 

In recent years, PGT-A techniques have continued to advance thanks to the implementation of high-resolution NGS-based methods. These approaches enable even more precise genetic assessment, allowing the detailed detection of chromosomal mosaicism and other genetic variants. Additionally, bioinformatics and data analysis have played a crucial role in the accurate interpretation of genomic analysis results. In the current era (PGT-A 3.0), NGS methodologies are being increasingly utilized. One of the applications of NGS in PGT-A is non-invasive preimplantation genetic testing (niPGT-A). This technique is based on the active secretion of DNA by the embryo into culture media at every stage of development, with enhanced secretion at the blastocyst stage. Cultured media containing this secreted DNA can be collected and analyzed using NGS. The great advantage of this new preimplantation diagnostic technique compared to those used until now is that it is non-invasive, meaning that it is not necessary to take cells from the embryo to carry out the diagnosis. Therefore, this avoids any potential harm that the embryo could experience during a biopsy. This approach was first applied in 2018 to understand the origin of this type of DNA and progressed to a prospective study in 2020 involving eight centers worldwide to evaluate the concordance between embryo cultured media analysis and TE biopsy: this study demonstrated that comparing TE biopsy and day 5 cultured media analysis resulted in 81.8% informativeness and 63% concordance [33]. However, analyzing the same samples on day 6 yielded 100% informativeness, reaching a conclusive analysis for both TE biopsy and cultured media with 84% concordance. Results significantly improved with additional steps aimed at enhancing the non-invasive analysis performance. One such step was introduced to prevent DNA contamination, involving embryo washing on day 4 before the day 6 collection, as embryos need 40 h in culture to secrete the DNA required for genetic investigation [34]. The reporting of results from the niPGT-A is unique, as it does not provide a clear aneuploidy diagnosis. It operates as a prioritization test, offering a percentage of embryo euploidy. The analysis software prioritizes certain embryos over others, especially those with higher euploidy scores. Therefore, this strategy may cause the exclusion of potentially euploid embryos in PGT-A.

### 4.1. Advantages and Limitations of PGT-A

Thanks to the possibility of selecting euploid embryos before transfer, PGT-A provides a higher likelihood of a successful pregnancy and a reduction in spontaneous miscarriages. The chromosomal analysis performed allows physicians to transfer single embryos, reducing the risk of multiple pregnancies. Among other advantages, there are time efficiency (providing rapid results, enabling embryo transfer in a short timeframe) and psychological benefits, as it can alleviate the emotional burden on couples by providing information about embryo viability. Like any other technique, there are also limitations. One of the most significant is mosaicism. This term refers to the presence of different cell lineages within an embryo. The limitation of PGT-A in this case is its inability to consistently detect mosaicism, thereby risking the transfer of an embryo with chromosomal abnormalities. Another important limitation is the impact of the procedure on the embryo, as the removal of some cells from the embryo may determine a slight impact on its viability. Finally, other limitations of the technique include the possibility of false positives and false negatives, ethical considerations, a cost increase in addition to in vitro fertilization, and the probability of inadvertently discarding viable embryos.

### 4.2. Mosaicism

Mosaicism refers to the presence of two or more cellular lineages with different genetic compositions within an individual. It originates from postzygotic mitotic errors, including errors in chromosomal segregation and postzygotic mitotic trisomy/monosomy and the rescue of meiotic origin aneuploidies. The reported incidence of mosaicism reaches up to 73% in cleavage-stage embryos, is reduced to 2–50% in blastocysts, and is further decreased during pregnancy to <0.2%, estimated in newborns [35]. Mosaicism occurs more frequently in the early stages of embryonic development and is a potential source of error in PGT-A. When mosaicism is present in an embryo, there is a risk that the biopsy may not represent the entire embryo [36]. PGT-A using FISH is not capable of detecting mosaicism, but the application of next-generation technologies such as NGS or aCGH in TE biopsy has shown a high rate of mosaicism detection. Initially, most laboratories and clinics equated mosaic embryos with aneuploid embryos, and they were generally discarded. In humans, the prevalence and developmental potential of mosaic embryos remain subject to intense debate. Mosaicism is generally not detected at increased rates of in vitro fertilization treatment, suggesting that aneuploid cells in mosaic preimplantation embryos do not contribute to the genetic composition of live-born infants [37]. A small study on mosaic embryos demonstrated their ability to give rise to healthy live births [38]. Retrospective studies have concluded that mosaic embryos have reduced reproductive potential [39]. However, such data are influenced by selection bias. In particular, analyses do not account for the fact that mosaic embryos are transferred as a last option and, consequently, their reproductive performance is often measured in a highly selected subpopulation of women who have experienced previous implantation failures.

To summarize, the history of PGT-A techniques reflects the constant advancement of genetic technologies and the progress of knowledge in the field of human genetics. From initial limited methods to sophisticated sequencing-based techniques, PGT-A has revolutionized the approach to embryo selection, opening new possibilities for couples seeking a healthy pregnancy. However, research and innovation continue to drive the field as professionals seek to address the remaining challenges and further improve the accuracy and accessibility of PGT-A techniques.

## 5. Preimplantation Genetic Testing for Structural Rearrangements (PGT-SR)

Preimplantation genetic testing for structural rearrangements (PGT-SR) has emerged as a promising technology in the field of reproductive medicine, allowing couples with chromosomal rearrangements to reduce the risk of transmitting genetic abnormalities to their offspring [11]. The most common structural chromosomal rearrangements include inversions, deletions, and duplications, but, most notably, translocations, which can be inherited or occur de novo. The presence of such alterations significantly increases the risk of generating chromosomally imbalanced embryos, regardless of the woman’s age.

Just like PGT-A, the first technique used for PGT-SR was FISH, with locus-specific probes and a resolution for small rearrangements <2 Mb. It required preclinical work-up and was time- and cost-intensive, not covering all 24 chromosomes. Today’s technology allows for the evaluation of the entire chromosomal complement of the couple, thus analyzing the chromosomes involved in the imbalance [40]. Various methods are employed for PGT-SR, including FISH, aCGH, and NGS. This type of genetic testing does not require preclinical setup and is mainly performed on embryonic biopsies collected at the cleavage or blastocyst stage [41,42]. However, as with any innovation, PGT-SR is not without limitations and challenges that require a thorough understanding to enhance its effectiveness and clinical applicability. Despite the advancement of high-resolution genetic sequencing techniques, the accuracy of diagnosing chromosomal abnormalities can vary based on the size and complexity of the rearrangements. Small chromosomal segments or intricate rearrangements might elude detection, jeopardizing the accuracy of the test and potentially leading to erroneous treatment decisions.

### Variability in Structural Rearrangements

Depending on the breakpoints’ positions in the involved chromosomes, the genetic consequences can vary widely. Some rearrangements might have no evident effects on the health of the future child, while others could lead to severe disabilities or pregnancy losses. The difficulty in predicting the precise clinical impact of a given rearrangement can complicate genetic counseling and reproductive decisions. As already mentioned, chromosomal rearrangements encompass different categories (such as balanced translocations, Robertsonian translocations, insertions, and inversions), each presenting a set of unique challenges in the context of PGT-SR [43]. They can also greatly vary in terms of complexity and the involved loci: some rearrangements might involve a limited number of chromosomes and regions, while others could encompass multiple chromosomes and chromosomal segments. Such diversity of rearrangements necessitates in-depth genetic analysis to identify the involved regions and assess the chromosome balance. The accurate identification of breakpoints in chromosomal rearrangements is crucial to understanding the extent of the involved segments and whether there is a loss or gain of genetic material. However, pinpointing breakpoints accurately can be complex, especially when involving repetitive or complex chromosomal regions. Errors in breakpoint identification can lead to the misinterpretation of PGT-SR results. Embryos with chromosomal rearrangements can display variability in the distribution of altered chromosomes within embryonic cells. Some embryos might be mosaic, with some cells carrying the rearrangement and others being normal. This variability makes accurately assessing the presence and balance of rearrangements a critical challenge, as it could influence the decision regarding embryo transferability. PGT-SR is not limited to assessing the embryos at hand, as it can also have implications for future generations. In cases where one parent is a carrier of a chromosomal rearrangement, careful evaluation of how the rearrangement can be transmitted to offspring is necessary [44]. Addressing variability in chromosomal rearrangements requires the use of advanced genetic analysis methodologies, such as array comparative genomic hybridization (aCGH) and next-generation sequencing (NGS). These techniques can help to accurately identify involved regions, breakpoints, and the presence of mosaicism. Additionally, involving expert geneticists in result interpretation is crucial in making informed decisions about embryo transferability.

In conclusion, variability in chromosomal rearrangements adds significant complexity to PGT-SR. The diversity of rearrangements, the challenge in identifying breakpoints, mosaic patterns, and transmission to the next generations demand thorough genetic assessment and careful planning. Furthermore, technological limitations in PGT-SR can influence result accuracy and reliability, potentially impacting couples’ reproductive decisions. Tackling these challenges requires the ongoing development of genetic sequencing technologies, careful result interpretation, and clear, comprehensive communication with the involved couples. Integrating new technological approaches and validation strategies can contribute to improving the clinical effectiveness of PGT-SR and ensuring informed and conscious choices for the families involved.

## 6. Innovative Approaches

Over the years, generic protocols have been developed for monogenic diseases and numerical and structural chromosomal abnormalities, allowing for the integration of PGT-M, PGT-A, and PGT-SR. Among these is the “One PGT solution”, which employs NGS (MDA protocol) to identify both monogenic diseases and numerical and structural chromosomal abnormalities.

Another strategy is “Haploseek”, which combines the CNV protocol (for the identification of alterations in PGT-A and PGT-SR) with SNP array (mainly focused on PGT-M).

**Figure 2:**
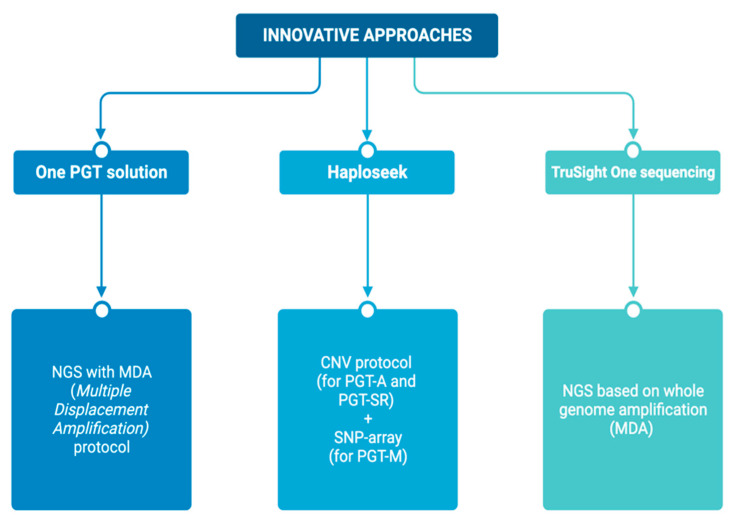
Innovative approaches of PGT.

The latest approach is “TruSight One sequencing”, an NGS approach that relies on whole-genome amplification (MDA) and does not require preclinical work-up because markers for approximately 5000 target genes are already included. This covers many genetic conditions, albeit with the limitation of requiring thorough validation before clinical use. Interpreting the results is also a complex process, as the analysis of the genome yields increasingly intricate information (Figure 2).

## 7. Conclusions

Preimplantation genetic diagnosis offers a unique opportunity to prevent hereditary diseases, but it is crucial to understand and address the challenges associated with these techniques. A critical assessment of the technical, ethical, and clinical limitations of PGD is essential for the informed and responsible use of past, current, and future techniques. Naturally, all diagnostic limitations must not only be carefully discussed with the patient during genetic counseling but also disclosed in the informed consent process. Continuous innovation and interdisciplinary collaboration among scientists, clinicians, and ethical experts are mandatory in tackling such challenges and maximizing the beneficial potential of PGD in clinical practice and medical research.

## Data Availability

No new data were created or analyzed in this study. Data sharing is not applicable to this article.
